# Bellevue Hospital Medical College

**Published:** 1871-06

**Authors:** 


### *Bellevue Hospital Medical College.*

We call special attention to the advertisement of the above Medical College, to be found in this number of the Journal. As will be seen from the advertisement, the Faculty embraces some of the first medical minds of this or any other country.

Although this is, comparatively, a new school, (having been organized in 1861, if we are not mistaken) it is composed of "live" and working men, and for years has ranked with the first Medical Colleges of this continent.

